# Personalizing cardiovascular risk prediction for patients with systemic lupus erythematosus

**DOI:** 10.1016/j.semarthrit.2024.152468

**Published:** 2024-05-17

**Authors:** May Y. Choi, Hongshu Guan, Kazuki Yoshida, Misti Paudel, Benjamin A. Kargere, Daniel Li, Jack Ellrodt, Emma Stevens, Tianrun Cai, Brittany N. Weber, Brendan M. Everett, Karen H. Costenbader

**Affiliations:** a Division of Rheumatology, Inflammation and Immunity, Brigham and Women’s Hospital and Harvard Medical School, Boston, MA, USA; b Division of Rheumatology, University of Calgary, Calgary, Alberta, Canada; c Williams College, Williamstown, MA, USA; d Division of Cardiovascular Medicine, Brigham and Women’s Hospital and Harvard Medical School, Boston, MA, USA; e Division of Preventive Medicine, Brigham and Women’s Hospital and Harvard Medical School, Boston, MA, USA

**Keywords:** Systemic lupus erythematosus, Cardiovascular risk, Prediction

## Abstract

**Objective:**

Cardiovascular disease (CVD) risk is increased in SLE and underestimated by general population prediction algorithms. We aimed to develop a novel SLE-specific prediction tool, SLECRISK, to provide a more accurate estimate of CVD risk in SLE.

**Methods:**

We studied patients in the Brigham and Women’s Hospital SLE cohort. We collected one-year baseline data including the presence of traditional CVD factors and SLE-related features at cohort enrollment. Ten-year follow-up for the first major adverse cardiovascular event (MACE; myocardial infarction (MI), stroke, or cardiac death) began at day +1 following the baseline period (index date). ICD-9/10 codes identified MACE were adjudicated by board-certified cardiologists. Least absolute shrinkage and selection operator regression selected SLE-related variables to add to the American College of Cardiology/American Heart Association (ACC/AHA) Pooled Cohort Risk Equations 10-year risk Cox regression model. Model fit statistics and performance (sensitivity, specificity, positive/negative predictive value, c-statistic) for predicting moderate/high 10-year risk (≥7.5 %) of MACE were assessed and compared to ACC/AHA, Framingham risk score (FRS), and modified FRS (mFRS). Optimism adjustment internal validation was performed using bootstrapping.

**Results:**

We included 1,243 patients with 90 MACEs (46 MIs, 36 strokes, 19 cardiac deaths) over 8946.5 person-years of follow-up. SLE variables selected for the new prediction algorithm (SLECRISK) were SLE activity (remission/mild vs. moderate/severe), disease duration (years), creatinine (mg/dL), anti-dsDNA, anti-RNP, lupus anticoagulant, anti-Ro positivity, and low C4. The sensitivity for detecting moderate/high-risk (≥7.5 %) of MACE using SLECRISK was 0.74 (95 %CI: 0.65, 0.83), which was better than the sensitivity of the ACC/AHA model (0.38 (95 %CI: 0.28, 0.48)). It also identified 3.4-fold more moderate/high-risk patients than the ACC/AHA. Patients who were moderate/high-risk according to SLECRISK but not ACC/AHA, were more likely to be young women with severe SLE and few other traditional CVD risk factors. Model performance between SLECRISK, FRS, and mFRS were similar.

**Conclusion:**

The novel SLECRISK tool is more sensitive than the ACC/AHA for predicting moderate/high 10-year risk for MACE and may be particularly useful in predicting risk for young females with severe SLE. Future external validation studies utilizing cohorts with more severe SLE are needed.

## Introduction

Systemic lupus erythematosus (SLE), a multi-system inflammatory autoimmune disease, accelerates atherosclerotic cardiovascular disease (CVD). Despite improvements in prevention and management over the past several decades, CVD remains the most common cause of mortality among patients with SLE [1–9]. Myocardial infarctions (MI) and strokes have an incidence at least 2 to 3-fold higher in SLE than in the general population, with increased risk among young patients and those with higher SLE disease severity [6,8–10]. Uncontrolled systemic inflammation and exposure to some SLE medications e.g., glucocorticoids, are among the important risk factors that have been identified as drivers of atherosclerosis in SLE [11,12].

Recognizing CVD as an important complication of SLE, several SLE guidelines recommend regular CVD risk assessments, including evaluation of traditional CVD risk factors [13–16]. However, general population models based on traditional risk factors alone including the Framingham Risk Score (FRS), the 2013 American College of Cardiology/American Heart Association (ACC/AHA) pooled cohort equation-based estimated risk, Systematic Coronary Risk Assessment (SCORE), and QRISK, do not fully explain the increased CVD risk among patients with SLE, likely because they do not capture SLE-specific risk factors, such as disease severity and treatment exposure [17–22]. For example, Esdaile et al. found that the FRS vastly underestimated CVD risk in SLE [19]. Jafri et al. confirmed that both FRS and ACC/AHA performed poorly in SLE [20]. Urowitz et al. attempted to address this by doubling the Framingham model (modified FRS or mFRS) estimates, which improved performance, but the mFRS was still insensitive for CVD risk prediction in SLE (sensitivity 32 %; specificity 81 %) [21]. The 2018 ACC/AHA Multispecialty Society Guideline on the Management of Blood Cholesterol recommended evaluation for “risk enhancers”, including chronic inflammatory conditions such as SLE and rheumatoid arthritis, to supplement the ACC/AHA to guide initiation of statin therapy for primary CVD prevention [23]. However, these inflammatory disease risk enhancers have not been assigned specific quantitative values for weighting risk estimates [23].

CVD risk prediction algorithms are important for estimating patients’ risk for developing future events and allow for more informed shared decision-making with patients. Accurately stratifying patients by risk group would allow for targeting interventions to those at the highest risk and potentially more effective healthcare delivery that can lower CVD morbidity and mortality. The lack of highly sensitive and specific CVD risk prediction algorithms for use in SLE creates uncertainty for patients and providers in estimating individualized risk. Better calibrated SLE CVD risk stratification is thus essential to clinical care, as well as to prevention studies, trial design, and interpretation. We sought to develop a better-performing SLE CVD risk prediction algorithm, accounting for SLE disease heterogeneity while remaining practical for use in a clinical setting.

## Methods

### Study cohort and design

**Fig. 1.**
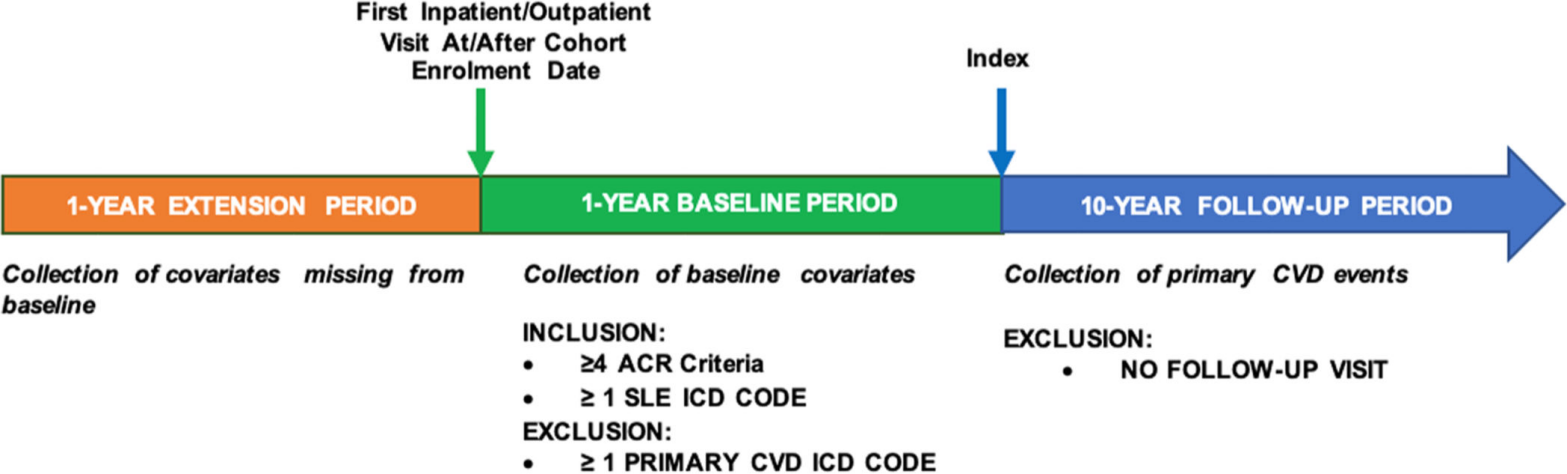
Study design. We identified patients with SLE based using ICD-9/10 codes and meeting the Updated 1997 American College of Rheumatology (ACR) SLE criteria without a history of major adverse cardiovascular event (MACE, myocardial infarction (MI), stroke, and cardiac death) from the electronic medical records (EMR). One-year baseline data including traditional cardiovascular disease (CVD) risk factors, demographics, and SLE-related clinical features were collected from the EMR at cohort enrollment. We allowed for a one-year extension period to collect covariate data that was missing from baseline. Ten-year follow-up for first MACE began at day +1 following the baseline period (index date). ICD-9/10 codes identified MACE adjudicated by medical record review by board-certified cardiologists.

We included SLE patients ≥18 years old, enrolled in the Brigham and Women’s Hospital SLE cohort between August 1995 and December 2019 with confirmed SLE and meeting the Updated 1997 American College of Rheumatology (ACR) SLE classification criteria [24,25]. All subjects were required to have one or more visits for SLE during the one-year baseline period (Fig. 1). A 10-year follow-up period for a major adverse cardiovascular event (MACE), a composite of non-fatal myocardial infarction, non-fatal stroke, and cardiovascular deaths, began one day following the baseline period (index date). We excluded those who did not have any follow-up visits (Supplemental Fig. 1). We also excluded subjects with MACE before the index date.

### Ascertainment of CVD events

The primary outcome was a MACE. The first MI or stroke in the follow-up period was identified using ICD-9/10 codes (Supplementary Table 1). These events were adjudicated by medical record review by board-certified cardiologists according to the Fourth Universal Definition of MI [26] by the Joint European Society of Cardiology (ESC)/ACC/AHA/World Heart Federation (WHF) Task Force and the 2013 Updated Definition of Stroke for the 21st Century by the AHA/American Stroke Association [27]. The causes of death were also adjudicated to rule out other causes of non-cardiac-related deaths.

### Ascertainment of potential predictors

We collected baseline data from the one-year before the index date on traditional CVD risk factors, and demographic and clinical features from the electronic health record at cohort enrollment (Fig. 1). We allowed a one-year extension period before the enrolment visit (the first visit of the baseline period) to allow for the collection of missing covariates. As traditional CVD risk factors are still important predictors of CVD risk among SLE patients, we used an existing CVD algorithm as the base model and added SLE-specific predictors to it. The base model selected was the 2013 ACC/AHA ten-year risk prediction score, a well-established and extensively validated algorithm. It includes age at baseline, sex, race, total cholesterol level, high-density lipoprotein (HDL) level, low-density lipoprotein (LDL) level, systolic blood pressure (SBP), diastolic blood pressure (DBP), treatment for hypertension, diabetes mellitus status, and smoking status (current) at baseline. In addition, it also accounts for sex (male/female) and race (White/Other/African American) by using different coefficients for each specific sex/race profile [28]. This represents an improvement over other risk scores such as the FRS [29], based on White patients only.

Baseline SLE-related variables included serum creatinine, SLE duration (in years), presence of lupus nephritis, physician global assessment of disease activity at the most recent baseline visit and over the entire baseline year (next section for details), serological status (antinuclear antibodies [ANA], antibodies to double-stranded DNA [dsDNA], ribonucleoprotein (RNP), Smith (Sm), Ro [anti-Ro60/SSA or anti-Ro52/Tripartite motif-containing protein 21 (TRIM21)], SSB/La, as well as lupus anticoagulant, anti-cardiolipin IgG and IgM, anti-β2glycoprotein 1 [β2GP1] IgG and IgM), low complement component 3 (C3) or complement component 4 (C4). It also included current SLE medications at baseline for SLE: oral and intravenous glucocorticoids, hydroxychloroquine, mycophenolate mofetil, cyclophosphamide, azathioprine, rituximab, cyclosporin, leflunomide, methotrexate, tacrolimus, intravenous immunoglobulin, and belimumab.

### Physician global assessment of disease activity

As SLE disease activity scores such as the Systemic Lupus Erythematosus Disease Activity Index 2000 (SLEDAI-2K, [30]) were not systematically assessed in clinical practice, SLE disease activity was retrospectively rated independently by two reviewers (KC and MC) by electronic health record review using a modified physician global assessment (PGA) tool. The PGA is designed to be relatively simple and intuitive to calculate so that the new CVD risk tool can be easily adopted into clinical practice. PGA scores ranged from 1 (remission/mild), 2 (moderate), to 3 (severe). The definition of each disease activity state was generated using the European League Against Rheumatism (EULAR) SLE management guideline [16] and the SLE severity algorithm developed for claims data [31] (Supplementary Table 2). Retrospective assessment of disease activity using the PGA has been previously validated [32]. Two PGA ratings were performed for each patient, one based on the last physician encounter before the study index date and one representing their overall disease activity for encounters during the entire baseline year. For training, the two reviewers completed PGAs for 100 of the same patients. Inter-rater agreement was 93.0 % for PGA of disease activity at the last visit prior to the index date and 93.5 % for overall disease activity. This corresponds to weighted kappas of 0.63 (standard error (SE) 0.08, *p* < 0.0001) and 0.75 (SE 0.08, *p* < 0.001), respectively.

### Statistical analysis

We assessed the baseline characteristics of the patients with SLE, comparing those with vs. without MACE in the follow-up period using *t*-tests, Chi-square tests, and Fisher exact tests. We then followed the Steyerberg Framework for developing and assessing the performance of our new CVD risk model, SLECRISK [33]. Our approach was to create a modification of the original ACC/AHA 10-year risk prediction model [28], by adding select SLE-related features and medications. We first applied the ACC/AHA equation to our SLE cohort to obtain the expected 10-year risk.

Least absolute shrinkage and selection operator (LASSO) regression was used to select the SLE features to be added to the ACC/AHA model. LASSO regression allows for both shrinkage and variable selection simultaneously for better prediction and model interpretation [34]. It performs automatic feature selection to decide which features to include and adds an L1 penalty (absolute values of the magnitude of the coefficients) to the loss function, encouraging the model to shrink coefficients to zero to prevent overfitting [34]. Cox regression models estimated the hazard of developing MACE in ten years of follow-up. We classified patients as low risk (<7.5 % 10-year MACE risk), moderate risk (7.5–20 %), and high risk (>20 %). We tested the Cox proportional hazard assumption for all selected variables in the model by generating time-dependent covariates by creating interactions of predictors and a function of survival time. We then tested the predictors and time-dependent covariates in the Cox model. If any of the time-dependent covariates are statistically significant, then the Cox proportional hazard assumption is not valid. Optimism adjustment internal validation was performed using bootstrapping with 300 samples as the recommended approach for prediction models by Steyenberg et al. [35,36]. We assessed model fit metrics with the Akaike Information Criterion (AIC) and Hosmer Lemeshow Chi-square statistic [37].

Using a cut-off of 7.5 % (<7.5 % low risk vs. ≥7.5 % as moderate/ high risk) as in past studies [38,39], we examined discrimination statistics and reclassification metrics comparing ACC/AHA model, FRS, and the mFRS to our model (SLECRISK) [21]. FRS includes age, sex, treatment for hypertension, diabetes status, smoking status, HDL cholesterol, total cholesterol and systolic blood pressure. mFRS for SLE applies a two-times multiplier to the FRS for a patient with an SLE diagnosis.

Results present sensitivity, specificity, positive predictive value, negative predictive value, c-statistic (receiver operating curves (ROC)) at year ten, net reclassification index (NRI), and integrated discrimination improvement (IDI). NRI for survival data is defined as the sum of (1) the proportion of risk score increased by the new model among 10-year cases and (2) the proportion of risk score decreased by the new model among 10-year event-free survivors [40]. IDI for survival data is defined as the sum of (1) the average predicted risk score increased by the new model among 10-year cases and (2) the average predicted risk score decreased by the new model among 10-year event-free survivors [41]. We obtained calibration plots for 10-year predictions for MACE comparing each model to SLECRISK.

We also compared the baseline characteristics of patients who would be considered at least moderate risk (7.5 % or greater) on the SLECRISK but not on the ACC/AHA model. As a sensitivity analysis, we analyzed the performance of the models using only the definite (adjudicated) MACE outcomes. In another analysis, we also modified the cut-off for low vs. moderate/high risk to <10 % vs. ≥10 % to reflect the cut-offs used in older studies [42].

For missing data, we performed the SAS PROC MI procedure to perform Fully Conditional Specification (FCS) multiple imputation. Analyses were conducted using SAS 9.4 (SAS Institute, Cary NC) and R V.4.1.2. We used the SAS macros to get the NRIs. We used the R package to get the IDIs at year 10.

## Results

### Baseline characteristics of participants

We included 1,243 patients with a total of 8946.51 person-years of follow-up. Our cohort was predominantly female (93.0 %) with a mean age of 41.62 (standard deviation, SD, 13.35) and a median duration of disease of 10.68 years (SD 9.02). 88.1 % of patients had remission or mild disease activity, 7.8 % had moderate, and 3.9 % had severe SLE activity at the visit prior to the follow-up period; 80.6 % of patients had remission or mild disease, 8.9 % had moderate, and 10.3 % had severe SLE over the entire baseline year.

We identified 90 MACEs, including 46 MIs, 36 strokes, and 19 cardiac deaths (Table 1). Patients with MACE had more traditional CVD risk factors compared to patients without (*n* = 1153), including older age (50.2 years vs. 41.0years), higher mean SBP (131.9 mmHg vs. 121.5 mmHg) and DBP (81.1 mmHg vs. 75.3 mmHg), and higher proportion of anti-hypertensive use (53.3 % vs. 31.5 %), smokers (20.0 % vs. 11.8 %), and diabetes (16.7 % vs. 6.2 %). Regarding SLE clinical features, patients with CVD had longer SLE disease duration (mean 15.4 years vs. 10.3 years) and more features of severe disease, including higher mean serum creatinine (1.42 mg/dL vs. 0.96 mg/dL), presence of autoantibodies (including anti-dsDNA, anti-Ro, and lupus anticoagulant), and hypocomplementemia (low C4). Among those with MACE, there was also a lower proportion of patients on hydroxychloroquine (52.2 % vs. 60.9 %).

### New SLECRISK equation

We identified eight SLE-related variables using LASSO regression (Table 2) and derived the following equation to predict 10-year MACE Risk:

**Table:** 

$\text{SLECRISK 10-Year MACE Risk}=1-0.92368^{\exp(\text{βx}-1.84311)}$
${\text{β}}_{\text{x}}={\text{β}}_{1}\text{ACC/AHA Risk Score}+{\text{β}}_{2}*\text{Disease Activity at Last Visit}+{\text{β}}_{3}*\text{Disease Duration}\;\;+{\text{β}}_{4}*\text{Creatinine}+{\text{β}}_{5}*\text{Anti-dsDNA}+{\text{β}}_{6}*\text{Anti-RNP}+{\text{β}}_{7}*\text{Lupus Anticoagulant}+\;\;{\text{β}}_{8}*\text{Anti-Ro}+{\text{β}}_{9}*\text{Low C4)}$
(Refer to Table 2 for β coefficient values)

### Comparison of SLECRISK performance to other models

**Fig. 2.**
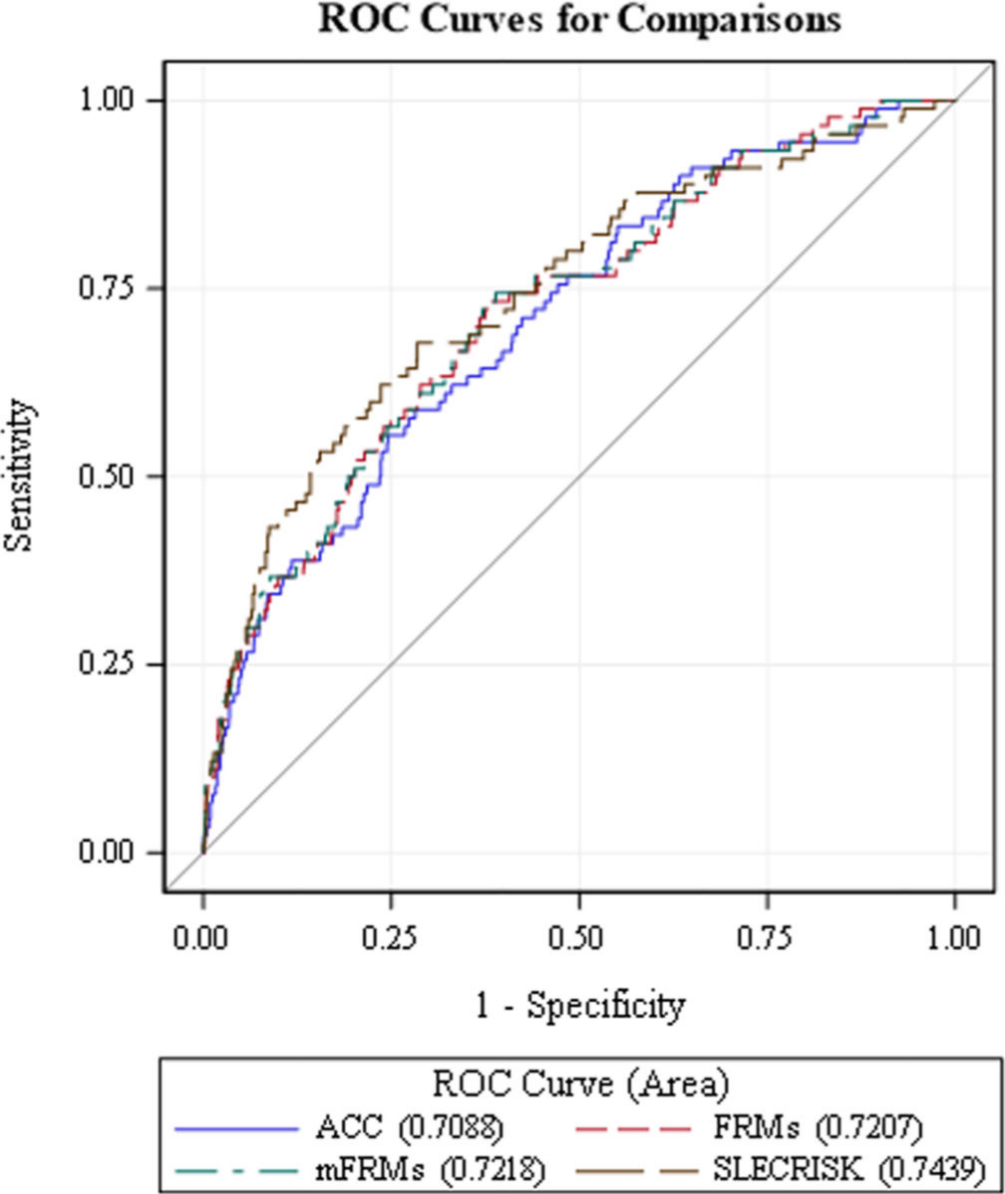
Receiver Operating Curves (ROC) at 10 years for novel SLECRISK compared to the American College of Cardiology/American Heart Association (ACC/AHA), Framingham Risk Score (FRS), and Modified Framingham Risk Score (mFRS) prediction models.

The Cox proportional assumption was valid for all selected variables. In predicting moderate-to high-risk (≥7.5 % 10-year MACE risk), SLECRISK had higher sensitivity (0.74 (95 % confidence interval (CI): 0.65, 0.83)) compared to ACC/AHA (0.38 (95 %CI: 0.28,0.48)) (Table 3, Fig. 2). The remainder of the performance metrics were similar between SLECRISK and ACC/AHA. There was also no difference between SLECRISK and FRS and mFRS in sensitivity, specificity, PPV, NPV, and Harrell’s c-statistics as confidence intervals overlapped. SLECRISK had the lowest AIC (592.18) and Hosmer-Lemeshow goodness-of-fit compared to the other models indicating good model calibration and best fit. There was a statistically significant positive IDI comparing SLECRISK and ACC/AHA [0.04 (0.01, 0.08) *p* = 0.02] indicating improved reclassification with SLECRISK.

**Fig. 3.**
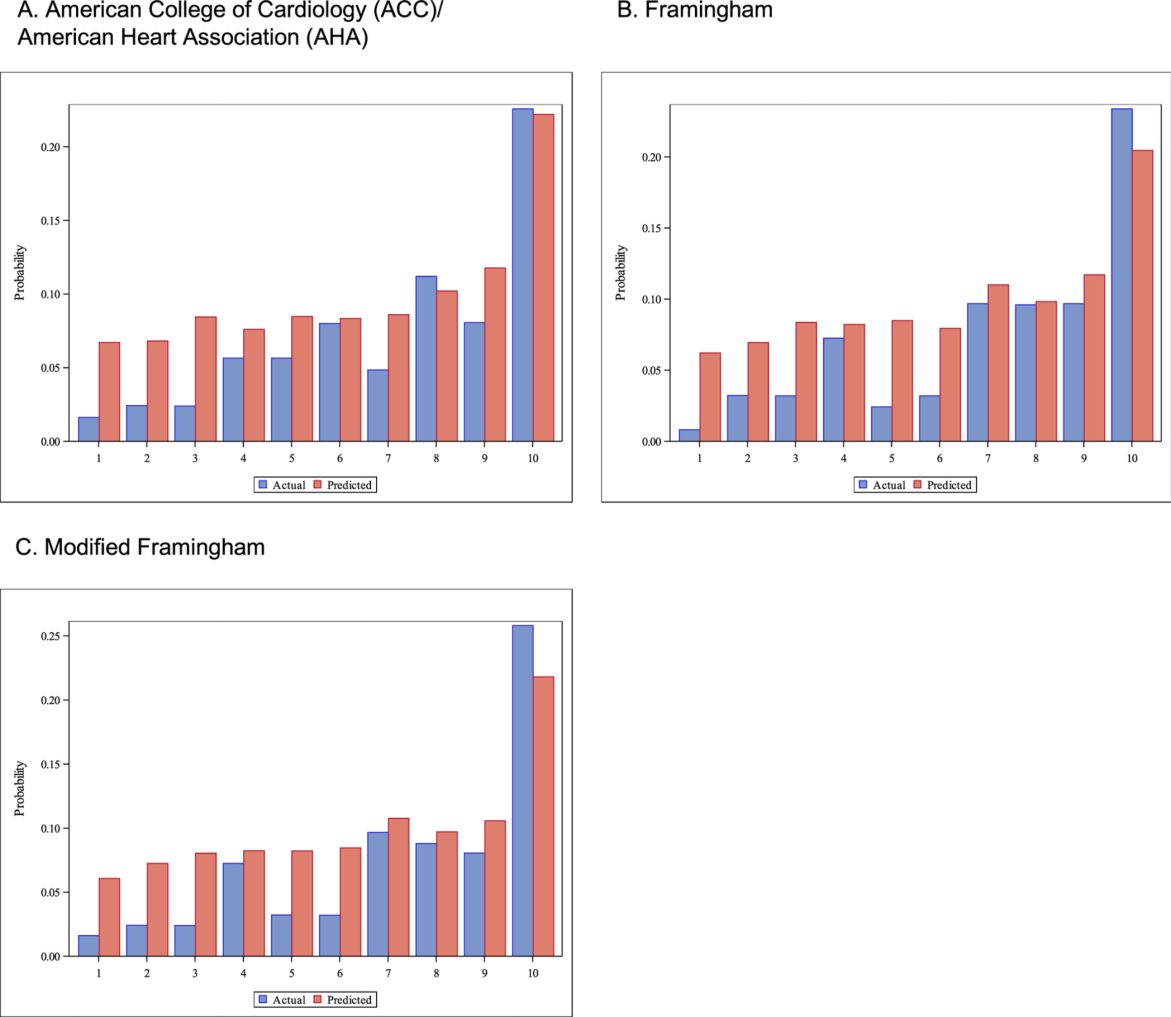
Calibration plots for ten-year predictions for Major Adverse Cardiovascular Event (MACE). X-axis is the deciles of the American College of Cardiology/American Heart Association (ACC/AHA) (A), Framingham Risk Score (FRS) (B), and modified Framingham Risk Score (mFRS) (C) models. Y-axis is the actual probability (observed definite and probable MACE event) and predicted probability (risk score from the SLECRISK formula).

In addition to looking at discrimination statistics, we assessed calibration by plotting the predicted versus observed risks for the SLECRISK model across deciles of predicted risk for original ACC/AHA, FRS, and mFRS (Fig. 3). In general, predicted risks were similar to observed risks across deciles for each of the risk prediction models, with a slight increase in risk prediction for the SLECRISK model.

### Risk classification

SLECRISK identified 4.2-fold more high-risk patients (>20 %) and 3.4-fold more moderate- and high-risk patients (≥7.5 %) than did the ACC/AHA model (Table 3). It also identified 1.2-fold more and approximately the same number of moderate- and high-risk patients than FRS and mFRS, respectively.

Of the 615 moderate-high-risk patients in the cohort classified by ACC/AHA and/or SLECRISK, only 124 (20.2 %) patients would have been classified as moderate-high-risk by both algorithms. 45 (7.3 %) patients would have been classified as moderate-high risk by ACC/AHA alone. By comparison, a larger proportion of patients (446 or 72.5 %) was classified in this category by SLECRISK only and would have been missed by ACC/AHA (Table 4). When comparing the baseline characteristics of the SLECRISK high-risk patients who would have been missed by the ACC/AHA algorithm (*n* = 446) to those who were classified as moderate-high risk by the ACC/AHA (*n* = 169), the former group of patients was younger with fewer CVD risk factors, as well as longer SLE disease duration, higher proportion of patients with lupus nephritis, autoantibodies (particularly antiphospholipid antibodies), hypocomplementemia, and more likely to be on mycophenolate mofetil, azathioprine, and statins.

### Sensitivity analysis

When the cut-off for moderate/high risk was changed from ≥7.5 % to ≥10 % the sensitivity of SLECRISK decreased to 0.64 (95 %CI: 0.55, 0.74). Relative to the ACC/AHA, the sensitivity of SLERISK remained higher [ACC/AHA, 0.27 (95 %CI: 0.18, 0.36)] (Supplemental Table 3). ACC/AHA had the highest specificity (0.94 (95 %CI: 0.92, 0.95)) followed by SLECRISK [0.72 (95 % CI: 0.70, 0.75). The NRI and IDI were positive and statistically significant for SLECRISK compared to ACC/AHA suggesting improved model discrimination with SLECRISK.

Most MACE outcomes (74/90, 82.2 %) were confirmed by board-certified cardiologists (i.e., definite events), including 26/46 MIs and 35/36 strokes. The remaining events did not meet enough criteria in the accepted definitions of MIs [26] and strokes [27] because of missing information in the electronic health records. When these were excluded, SLECRISK still demonstrated a higher sensitivity of 0.76 (95 %CI: 0.66, 0.85) vs. 0.38 (95 %CI: 0.27, 0.49) and 0.64 (95 %CI: 0.53, 0.74) vs. 0.26 (95 %CI: 0.16, 0.36) compared to ACC/AHA at the 7.5 % and 10 % cut-off respectively (Supplemental Tables 4 and 5). When we analyzed only the definite (adjudicated) MACE at the 10 % cut-off, the results were similar to the analysis that considered both definite and probable MACE at the same cut-off (Supplemental Table 6).

## Discussion

Recognizing that current prediction models developed for the general population greatly underestimate CVD risk for patients with SLE, our goal was to develop a novel, sensitive, and practical disease-specific tool, SLECRISK. SLECRISK was developed to enhance the detection of moderate- to high-risk for MACE among patients with SLE with greater sensitivity, thereby reducing the likelihood that these patients are overlooked for preventive and risk-modifying therapy. SLECRISK is an extension of the widely recommended ACC/AHA model, which incorporates sex and race, representing an important methodological improvement over other risk scores such as FRS that are based predominantly on observations in White patients.

We were able to demonstrate that SLECRISK was a more sensitive tool than the traditional ACC/AHA model (74 % vs. 38 %) and reclassified more moderate/high-risk patients correctly into their elevated risk category. It detected 3.4-fold more moderate/high-risk patients and was particularly useful in identifying young SLE patients with severe disease activity who otherwise have few traditional CVD risk factors. As an example: A 50-year-old woman with SLE has a 5 % ACC/AHA risk score (low risk). However, she also has high recent SLE disease activity, ten years of disease duration, elevated serum creatinine (2.26 mg/dL), is anti-dsDNA positive, and has a low C4. SLECRISK = 1–0.92^exp(βx−1.84)^_=_ 0.113 or 11.3 % (moderate risk). Therefore, her risk classification changed from low to moderate risk using the SLECRISK equation. The “low risk” of 5% using ACC/AHA likely would have been to discuss initiating primary prevention lipid-lowering therapy given the presence of SLE as a “risk enhancer”. On the other hand, the recommendation for “moderate risk” (≥7.5%−20%) using SLECRISK would be to start the patient on lipid-lowering (statins) therapy and/or undergo coronary artery calcium (CAC) imaging in shared-decision making about her increased risk [43]. CAC also has a role in determining whether patients with moderate CVD risk such as this might benefit from aspirin therapy for primary CVD prevention [44].

Importantly, the patient’s SLECRISK score suggests that they would also benefit from more aggressive control of their SLE disease activity. The elevated CVD risk in SLE patients has been attributed in large part to its pro-inflammatory state [45] and this is reflected in the SLE-related variables that were selected for SLECRISK: increased disease duration, disease activity, renal dysfunction, positive anti-dsDNA, -RNP,- Ro antibodies, lupus anticoagulant, and low C4. These are in keeping with other studies that have shown that these SLE factors are associated with increased CVD risk [1,6,46–49].

In another single-centre analysis of a similar SLE cohort to ours (predominantly White, young, female), Sivakumaran et al. also showed that the mFRS and other SLE CVD models (QRESEARCH risk estimator version 3 or QRISK3 [50] [22 variables], and SLECRE [51]) did not have greater accuracy (c-statistic 0.67–0.73) over the traditional tools (FRS, QRISK2 [52]) (c-statistic 0.72–0.73) [38]. This suggests a need for better CVD risk assessment tools for SLE population. While we could not directly compare our tool to other SLE CVD tools such as QRISK3 and SLECRE that collected other variables not included in our dataset (e.g., erectile dysfunction, severe mental illness, atypical antipsychotic use, the SELENA revision of the SLE Disease Activity Index [SELENA-SLEDAI] score [53]), we would expect that SLECRISK would have at least comparable performance. Rather, an important distinction of SLECRISK is that we specifically considered many more SLE-related factors that are routinely available and easily assessed (e.g., physician global assessment versus SLEDAI-2K) [54]. In comparison, other tools such as the Predictors of Risk for Elevated Flares, Damage Progression and Increased Cardiovascular Disease in Patients with SLE (PREDICTS) [55] and the Adjusted Global APSCVD Score (aGAPSS_CVD_) [56] are less feasible to calculate because they include biomarkers that are not routinely tested. We posit that the SLECRISK is more user-friendly and accessible for ease of clinical adoption.

We recognize that there are important limitations to this new model. This is a single-centre cohort of patients, the majority of whom were White with well-controlled disease activity. We performed internal validation of SLECRISK using bootstrap optimism correction and will be externally validating our tool in other and more diverse SLE cohorts. We were also not able to confirm that all the CVD events were definite events due lack of information in electronic health records, although the majority (82 %) were adjudicated by board-certified cardiologists as definite and sensitivity analysis demonstrated similar model performance using only adjudicated events. We did not use a competing risk model to avoid overestimating CVD risk and to allow for a more representative model of overall mortality risk in the SLE population. There were relatively few MACE outcomes in our study cohort, which is likely related to most patients (>78 %) having SLE disease that was well-controlled. While the AUCs for SLECRISK, ACC/AHA, FRS, and mFRS were otherwise similar in this cohort of predominantly well-controlled SLE patients, we hypothesize that more sensitive detection of risk using SLECRISK may result in more accurate classification in an SLE population with more severe disease where there is a higher prevalence of SLE-specific factors.

In conclusion, we derived and internally validated a novel SLE-specific CVD risk tool based on the ACC/AHA score that adds readily available and clinically relevant SLE-related variables. SLECRISK was more sensitive than traditional CVD tools for the general population in predicting moderate- and high-risk for MACE over ten years of follow-up. It offers advantages over other CVD SLE tools including inclusion of risk factors that are routinely collected and assessed in a physician’s office and may be a useful tool for accurately classifying higher risk among certain SLE groups, especially young females with severe SLE activity. With a risk prediction tool that is more tailored towards a SLE disease profile, clinicians may find this helpful to address SLE-related factors that may be driving long-term CVD. If externally validated, SLECRISK may be incorporated into SLE management guidelines to help guide early decision-making in the primary prevention of CVD in clinical practice.

## Supplementary Material

Figures and Tables with Supplemental Files

## Figures and Tables

**Table 1:** Baseline demographic, cardiovascular, and clinical features among 1,243 patients with SLE in the Brigham and Women’s Hospital Lupus Cohort with vs. without Major Adverse Cardiovascular Event (MACE)^a^ in follow-up.

	CVD Event (*n* = 90)	No CVD Event (*n* = 1153)	*P*-values

Demographics, mean (SD)			
Age	50.2 (14.0)	41.0 (13.1)	<0.0001
Female, *n* (%)	82 (91.1)	1074 (93.2)	0.47
White, *n* (%)	60 (66.7)	717 (62.2)	0.40
CVD Traditional Risk Factors			
Total Cholesterol, mg/dL, mean (SD)	189.5 (38.1)	182.2 (60.2)	0.20
HDL, mg/dL, mean (SD)	56.4 (18.7)	55.7 (20.0)	0.83
LDL, mg/dL, mean (SD)	110.5 (35.7)	99.2 (39.9)	0.09
SBP, mg/dL, mean (SD)	131.9 (28.2)	121.5 (16.5)	<0.001
DBP, mg/dL, mean (SD)	81.1 (16.7)	75.3 (11.0)	0.002
Anti-hypertensive, *n* (%)	48 (53.3)	363 (31.5)	<0.0001
Current Smoker, *n* (%)	18 (20.0)	136 (11.8)	0.02
Body Mass Index, kg/m^b^, mean (SD)	27.8 (6.1)	29.6 (40.0)	0.36
Diabetes, *n* (%)	15 (16.7)	71 (6.2)	<0.001
SLE Clinical Features			
Creatinine, mg/dL, mean (SD)	1.4 (1.5)	1.0 (1.0)	0.005
SLE Duration, mg/dL, mean (SD)	15.4 (11.6)	10.3 (8.7)	<0.0001
Lupus nephritis, *n* (%)	35 (38.9)	359 (31.1)	0.13
Physician Global Assessment most recent, *n* (%)			
Remission/Mild	74 (82.2)	1022 (88.6)	0.11
Moderate	9 (10.0)	88 (7.6)	
Severe	7 (7.8)	43 (3.7)	
Physician Global Assessment over the past year, *n* (%)			
Remission/Mild	70 (77.8)	933 (80.9)	0.52
Moderate	11 (12.2)	100 (8.7)	
Severe	9 (10.0)	120 (10.4)	
Positive Serologies and Low Complement Levels, *n* (%)			
Antinuclear antibody	87 (96.7)	1127 (97.8)	0.46
Anti-dsDNA	72 (80.0)	782 (67.8)	0.02
Anti-RNP	41 (45.6)	444 (38.5)	0.19
Anti-Sm	30 (33.3)	365 (31.7)	0.74
Anti-Ro	52 (57.8)	542 (47.0)	0.05
Anti-SSB/La	30 (33.3)	321 (27.8)	0.26
Lupus anticoagulant, *n* (%)	15 (16.7)	117 (10.2)	0.05
Anti-cardiolipin IgG, *n* (%)	22 (24.4)	236 (20.5)	0.37
Anti-cardiolipin IgM, *n* (%)	11 (12.2)	163 (14.1)	0.61
Anti-β2GP1 IgG, *n* (%)	6 (6.7)	74 (6.4)	0.93
Anti-β2GP1 IgM, *n* (%)	0 (0)	33 (2.9)	0.17
Any positive antiphospholipid, *n* (%)	24 (26.7)	270 (23.4)	0.48
C3, *n* (%)	44 (48.9)	526 (45.6)	0.55
C4, *n* (%)	41 (45.6)	369 (32.0)	0.01
Current Medications, *n* (%)			
Glucocorticoids^c^	57 (63.3)	650 (56.4)	0.20
Hydroxychloroquine	47 (52.2)	702 (60.9)	0.11
Mycophenolate mofetil	12 (13.3)	167 (14.5)	0.76
Cyclophosphamide	3 (3.3)	46 (4.0)	1.00
Azathioprine	12 (13.3)	151 (13.1)	0.95
Rituximab	1 (1.1)	21 (1.8)	1.00
Cyclosporin	1 (1.1)	10 (0.9)	0.56
Leflunomide	3 (3.3)	15 (1.3)	0.14
Methotrexate	2 (2.2)	91 (7.9)	0.05
Tacrolimus	1 (1.1)	18 (1.6)	1.00
IVIG	1 (1.1)	25 (2.2)	1.00
Belimumab	0 (0)	16 (1.4)	0.62
Aspirin	20 (22.2)	147 (12.8)	0.01
Statin	24 (26.7)	119 (10.3)	<0.0001
Warfarin	17 (18.9)	68 (5.9)	<0.0001
Angiotensin-converting enzyme (ACE) inhibitors	24 (26.7)	202 (17.5)	0.03
Angiotensin receptor blockers (ARBs)	14 (15.6)	46 (4.0)	<0.0001
Calcium channel blockers	26 (28.9)	136 (11.8)	<0.0001
Beta-blockers	32 (35.6)	141 (12.2)	<0.0001
Diuretics	5 (5.6)	65 (5.6)	0.97

a Major adverse cardiovascular event includes non-fatal myocardial infarction, non-fatal stroke, and cardiac death. This includes events adjudicated (definite) by board-certified cardiologists and probable events.

b Includes oral and intravenous glucocorticoids.

c *Abbreviations*: β2GP1, beta2 glycoprotein 1; c3, complement component 3; c4, complement component 4; CVD, cardiovascular disease; DBP, diastolic blood pressure; dsDNA, anti-double stranded DNA; HDL, high-density lipoprotein; IVIG, intravenous immunoglobulin; LDL, low-density lipoprotein; RNP, ribonucleoprotein; SBP, systolic blood pressure; SD, standard deviation; Sm, Smith; SLE, systemic lupus erythematosus.

**Table 2:** Beta-coefficients for predicting 10-year risk of Major Adverse Cardiovascular Event (MACE)^a^ among 1243 patients with SLE using SLECRISK and the American College of Cardiology/American Heart Association (ACC/AHA) risk score alone.

Variable		Beta-coefficients

ACC/AHA risk score only		
ACC/AHA	Without SLE variables	6.42
SLECRISK		
ACC/AHA	With SLE variables	5.44
SLE Disease Activity^b^	Remission/mild vs. moderate/severe	0.31
SLE Disease Duration	Years	0.04
Serum Creatinine	mg/dL	0.17
Anti-dsDNA	Ever positive at baseline	0.35
Anti-RNP	Ever positive at baseline	0.22
Lupus Anticoagulant	Ever positive at baseline	0.47
Anti-Ro	Ever positive at baseline	0.31
Low C4	Ever low at baseline	0.49

a Definite (adjudicated) and probable MACE were included. Beta coefficients derived from Cox regression model estimating the hazard of developing MACE in ten years of follow-up.

b Based on Physician Global Assessment of disease activity at the last visit prior to the index date (one day following the baseline period).

*Abbreviations*: C4, complement 4; dsDNA, anti-double stranded DNA; RNP, ribonucleoprotein; SLE, systemic lupus erythematosus.

**Table 3:** Novel SLECRISK, American College of Cardiology/American Heart Association (ACC/AHA), Framingham Risk Score (FRS), Modified Framingham Risk Score (mFRS) Model Performances for Prediction of <7.5 % (low risk) vs. ≥7.5 % (moderate/high risk) 10-Year Risks of Major Adverse Cardiovascular Event (MACE)^a^ among 1,243 Patients with SLE at Baseline.

	SLECRISK	ACC/AHA	FRS	mFRS

Low Risk (<7.5 %), *n* (%)	673 (54.1)	1074 (86.4)	781(62.8)	693 (55.8)
Moderate Risk (7.5–20 %), *n* (%)	456 (36.7)	142 (11.4)	313 (25.2)	205 (16.5)
High Risk (>20 %), *n* (%)	114 (9.2)	27 (2.2)	149 (12.0)	345 (27.8)
Sensitivity (95 %CI)	0.74 (0.65, 0.83)	0.38 (0.28, 0.48)	0.67 (0.57, 0.76)	0.74 (0.65, 0.83)
Specificity (95 %CI)	0.56 (0.54, 0.59)	0.88 (0.86, 0.90)	0.65 (0.62, 0.68)	0.58 (0.55, 0.61)
Positive Predictive Value (95 %CI)	0.12 (0.09, 0.14)	0.20 (0.14, 0.26)	0.13 (0.10, 0.16)	0.12 (0.09, 0.15)
Negative Predictive Value (95 %CI)	0.97 (0.95, 0.98)	0.95 (0.93, 0.96)	0.96 (0.95, 0.98)	0.97 (0.95, 0.98)
c-statistic (95 %CI) *p*-value for SLECRISK vs. Each Risk Score	0.74 (0.69, 0.80) *p* = reference	0.71 (0.65, 0.76) *p* = 0.28	0.72 (0.67, 0.78) *p* = 0.49	0.72 (0.67, 0.78) *p* = 0.51
AIC	592.18	617.81	595.78	595.35
Hosmer Lemeshow Chi-square statistic (*p*-value)	11.97 (0.15)	17.58 (0.02)	13.23 (0.10)	14.41 (0.07)
Net Reclassification Index (95 %CI) *p*-value for SLECRISK vs. Each Risk Score	–	0.05 (− 0.08, 0.19) *p* = 0.42	0.003 (− 0.10, 0.10) *p* = 0.95	− 0.006 (− 0.11, 0.09) *p* = 0.90
Integrated discrimination improvement (95 % CI) *p*-value for SLECRISK vs. Each Risk Score	–	0.04 (0.01, 0.08) *p* = 0.02	0.01 (− 0.04, 0.06) *p* = 0.81	0.001 (− 0.06, 0.06) *p* = 1.02

a Definite (adjudicated) and probable MACE.

*Abbreviations*: AIC, Akaike’s information criterion; AUC, area under the curve; ACC/AHA American College of Cardiology/American Heart Association; CI, confidence interval; FRS, Framingham Risk Score; mFRS, modified Framingham Risk Score.

**Table 4:** Comparison of Baseline Characteristics Between Moderate and High-Risk Patients (7.5 % and greater) for 10-Year Risks of Major Adverse Cardiovascular Event (MACE)^a^ among 1,243 Patients with SLE at Baseline using the American College of Cardiology/American Heart Association (ACC/AHA) Score vs. SLECRISK.

Baseline characteristics*	SLECRISK (*n* = 446)	ACC/AHA (*n* = 169)	*P*-value*

Demographics, mean (SD)			
Age	41.3 (11.4)	53.9 (17.6)	<0.0001
Female, *n* (%)	419 (94.0)	145 (85.8)	0.001
CVD Traditional Risk Factors, mean (SD)			
Total Cholesterol, mg/dL	182.9 (59.5)	191.8 (41.3)	0.04
LDL, mg/dL	101.3 (30.0)	107.5 (27.6)	0.02
SBP, mmHg	121.2 (16.5)	137.8 (22.7)	<0.0001
DBP, mmHg	75.9 (11.6)	80.3 (13.5)	0.0003
Anti-Hypertensive, *n* (%)	182 (40.8)	94 (55.6)	0.001
Smoking, *n* (%)	54 (12.1)	40 (23.7)	0.0004
Diabetes, *n* (%)	24 (5.4)	35 (20.7)	<0.0001
SLE Variables, mean (SD)			
Mean SLE Duration (SD)	15.0 (9.8)	12.2 (11.2)	0.005
Lupus nephritis, *n* (%)	198 (44.4)	57 (33.7)	0.02
Autoantibodies, *n*			
Anti-dsDNA	387 (86.8)	118 (69.8)	<0.0001
Anti-RNP	252 (56.5)	55 (32.5)	<0.0001
Anti-Sm	205 (46.0)	49 (29.0)	0.0001
Anti-SSB/La	186 (41.7)	39 (23.1)	<0.0001
Anti-Ro	309 (69.3)	78 (46.2)	<0.0001
Any Antiphospholipid Ab	131 (29.4)	34 (20.1)	0.02
Lupus anticoagulant	97 (21.8)	11 (6.5)	<0.0001
Anti-cardiolipin IgG	137 (30.7)	27 (16.0)	0.0002
Anti-β2 Glycoprotein I IgG	46 (10.3)	7 (4.1)	0.01
Anti-β2 Glycoprotein I			
IgM	20 (4.5)	2 (1.2)	0.049
Low C3, *n* (%)	304 (68.2)	68 (40.2)	<0.0001
Low C4, *n* (%)	270 (60.5)	50 (29.6)	<0.0001
Medications, *n* (%)			
Mycophenolate mofetil	89 (20.0)	22 (13.0)	0.046
Azathioprine	82 (18.4)	15 (8.9)	0.004
Statin	64 (14.4)	38 (22.5)	0.02

* Only statistically significant variables are shown (*p* < 0.05). Refer to Table 1 for the baseline variables that were analyzed.

a Definite (adjudicated) and probable major adverse cardiovascular event (MACE).

*Abbreviations*: Ab, autoantibody; ACC/AHA, American College of Cardiology/American Heart Association; C3, complement 3; C4, complement 4; CVD, cardiovascular disease; dsDNA, double-stranded DNA; HDL, high-density lipoprotein; Ig, Immunoglobulin; LDL, low-density lipoprotein; RNP, ribonucleoprotein; SBP, systolic blood pressure; SLE, systemic lupus erythematosus; Sm, Smith.

## Data Availability

Data from this project can be considered for release if the appropriate IRB and publication clearances have been made.
