# Supplementary material for: Personalizing cardiovascular risk prediction for patients with systemic lupus erythematosus
Source: Semin Arthritis Rheum. Author manuscript; Available in PMC 2025 Aug 1. (PMC11214838; doi:10.1016/j.semarthrit.2024.152468)
Supplement: Figures and Tables with Supplemental Files [file NIHMS2000699-supplement-Figures_and_Tables_with_Supplemental_Files.docx]

**TABLES AND FIGURES**

**Main Manuscript**

**Table 1. Baseline characteristics Definite and Probable MACE**

**Table 2. Beta Coefficients for Definite and Probable MACE**

**Table 3. Model Performance for Definite and Probable MACE at 7.5% cut-off**

**Table 4. Comparing High Risk Patients for Definite and Probable MACE at 7.5% cut-off**

**Figure 1. Study Design**

**Figure 2. ROC Logistic Regression Curves at Year 10**

**Figure 3. Calibration plots for ten-year predictions for MACE**

**Supplementary Materials**

**Table 1. ICD codes**

**Table 2. Physician Global Assessment Definition**

**Table 3. Model Performance for Definite and Probable MACE at 10% cut-off**

**Table 4. Beta Coefficients for Definite MACE Only**

**Table 5. Model Performance for Definite MACE Only at 7.5% cut-off**

**Table 6. Model Performance for Definite MACE Only at 10% cut-off**

**Figure 1. Cohort Inclusion/Exclusion Criteria**

**Table 1. Baseline demographic, cardiovascular, and clinical features among 1,243 patients with SLE in the Brigham and Women’s Hospital Lupus Cohort with vs. without Major Adverse Cardiovascular Event (MACE)^1^ in follow-up**

|  | **CVD Event (n=90)** | **No CVD Event (n=1153)** | **P-values** |
| --- | --- | --- | --- |
| **Demographics, mean (SD)** | | | |
| Age | 50.2 (14.0) | 41.0 (13.1) | <.0001 |
| Female, n (%) | 82 (91.1) | 1074 (93.2) | 0.47 |
| White, n (%) | 60 (66.7) | 717 (62.2) | 0.40 |
| **CVD Traditional Risk Factors** | | | |
| Total Cholesterol, mg/dL, mean (SD) | 189.5 (38.1) | 182.2 (60.2) | 0.20 |
| HDL, mg/dL, mean (SD) | 56.4 (18.7) | 55.7 (20.0) | 0.83 |
| LDL, mg/dL, mean (SD) | 110.5 (35.7) | 99.2 (39.9) | 0.09 |
| SBP, mg/dL, mean (SD) | 131.9 (28.2) | 121.5 (16.5) | <0.001 |
| DBP, mg/dL, mean (SD) | 81.1 (16.7) | 75.3 (11.0) | 0.002 |
| Anti-hypertensive, n (%) | 48 (53.3) | 363 (31.5) | <.0001 |
| Current Smoker, n (%) | 18 (20.0) | 136 (11.8) | 0.02 |
| Body Mass Index, kg/m^2^, mean (SD) | 27.8 (6.1) | 29.6 (40.0) | 0.36 |
| Diabetes, n (%) | 15 (16.7) | 71 (6.2) | <.001 |
| **SLE Clinical Features** | | | |
| Creatinine, mg/dL, mean (SD) | 1.4 (1.5) | 1.0 (1.0) | 0.005 |
| SLE Duration, mg/dL, mean (SD) | 15.4 (11.6) | 10.3 (8.7) | <0.0001 |
| Lupus nephritis, n (%) | 35 (38.9) | 359 (31.1) | 0.13 |
| Physician Global Assessment most recent, n (%) | | | |
| Remission/Mild | 74 (82.2) | 1022 (88.6) | 0.11 |
| Moderate | 9 (10.0) | 88 (7.6) |  |
| Severe | 7 (7.8) | 43 (3.7) |  |
| Physician Global Assessment over the past year, n (%) | | | |
| Remission/Mild | 70 (77.8) | 933 (80.9) | 0.52 |
| Moderate | 11 (12.2) | 100 (8.7) |  |
| Severe | 9 (10.0) | 120 (10.4) |  |
| Positive Serologies and Low Complement Levels, n (%) | | | |
| Antinuclear antibody | 87 (96.7) | 1127 (97.8) | 0.46 |
| Anti-dsDNA | 72 (80.0) | 782 (67.8) | 0.02 |
| Anti-RNP | 41 (45.6) | 444 (38.5) | 0.19 |
| Anti-Sm | 30 (33.3) | 365 (31.7) | 0.74 |
| Anti-Ro | 52 (57.8) | 542 (47.0) | 0.05 |
| Anti-SSB/La | 30 (33.3) | 321 (27.8) | 0.26 |
| Lupus anticoagulant, n (%) | 15 (16.7) | 117 (10.2) | 0.05 |
| Anti-cardiolipin IgG, n (%) | 22 (24.4) | 236 (20.5) | 0.37 |
| Anti-cardiolipin IgM, n (%) | 11 (12.2) | 163 (14.1) | 0.61 |
| Anti-β2GP1 IgG, n (%) | 6 (6.7) | 74 (6.4) | 0.93 |
| Anti-β2GP1 IgM, n (%) | 0 (0) | 33 (2.9) | 0.17 |
| Any positive antiphospholipid, n (%) | 24 (26.7) | 270 (23.4) | 0.48 |
| C3, n (%) | 44 (48.9) | 526 (45.6) | 0.55 |
| C4, n (%) | 41 (45.6) | 369 (32.0) | 0.01 |
| **Current Medications, n (%)** | | | |
| Glucocorticoids^3^ | 57 (63.3) | 650 (56.4) | 0.20 |
| Hydroxychloroquine | 47 (52.2) | 702 (60.9) | 0.11 |
| Mycophenolate mofetil | 12 (13.3) | 167 (14.5) | 0.76 |
| Cyclophosphamide | 3 (3.3) | 46 (4.0) | 1.00 |
| Azathioprine | 12 (13.3) | 151 (13.1) | 0.95 |
| Rituximab | 1 (1.1) | 21 (1.8) | 1.00 |
| Cyclosporin | 1 (1.1) | 10 (0.9) | 0.56 |
| Leflunomide | 3 (3.3) | 15 (1.3) | 0.14 |
| Methotrexate | 2 (2.2) | 91 (7.9) | 0.05 |
| Tacrolimus | 1 (1.1) | 18 (1.6) | 1.00 |
| IVIG | 1 (1.1) | 25 (2.2) | 1.00 |
| Belimumab | 0 (0) | 16 (1.4) | 0.62 |
| Aspirin | 20 (22.2) | 147 (12.8) | 0.01 |
| Statin | 24 (26.7) | 119 (10.3) | <.0001 |
| Warfarin | 17 (18.9) | 68 (5.9) | <.0001 |
| Angiotensin-converting enzyme (ACE) inhibitors | 24 (26.7) | 202 (17.5) | 0.03 |
| Angiotensin receptor blockers (ARBs) | 14 (15.6) | 46 (4.0) | <.0001 |
| Calcium channel blockers | 26 (28.9) | 136 (11.8) | <.0001 |
| Beta-blockers | 32 (35.6) | 141 (12.2) | <.0001 |
| Diuretics | 5 (5.6) | 65 (5.6) | 0.97 |
| 1. Major adverse cardiovascular event includes non-fatal myocardial infarction, non-fatal stroke, and cardiac death. This includes events adjudicated (definite) by board-certified cardiologists and probable events. 2. Includes oral and intravenous glucocorticoids 3. Abbreviations: β2GP1CVD, beta2 glycoprotein 1; c3, complement 3; c4, complement 4; cardiovascular disease; DBP, diastolic blood pressure; dsDNA, anti-double stranded DNA; HDL, high density lipoprotein; IVIG, intravenous immunoglobulin; LDL, low-density lipoprotein; SBP, systolic blood pressure; SD, standard deviation; SLE, systemic lupus erythematosus | | | |

**Table 2. Beta-Coefficients for Predicting 10-year risk of MACE^1^ among 1243 Patients with SLE using SLECRISK and the American College of Cardiology/American Heart Association (ACC/AHA) Risk Score Alone**

| **Variable** |  | **Beta-Coefficients** |
| --- | --- | --- |
| ACC/AHA risk score only | |  |
| ACC/AHA | Without SLE variables | 6.42 |
| SLECRISK | |  |
| ACC/AHA | With SLE variables | 5.44 |
| SLE Disease Activity^2^ | Remission/mild vs. moderate/severe | 0.31 |
| SLE Disease Duration | Years | 0.04 |
| Serum Creatinine | mg/dL | 0.17 |
| Anti-dsDNA | Ever positive at baseline | 0.35 |
| Anti-RNP | Ever positive at baseline | 0.22 |
| Lupus Anticoagulant | Ever positive at baseline | 0.47 |
| Anti-Ro | Ever positive at baseline | 0.31 |
| Low C4 | Ever low at baseline | 0.49 |
| 1. Definite (adjudicated) and probable MACE were included. Beta coefficients derived from Cox regression model estimating the hazard of developing MACE in ten years of follow-up.  2. Based on Physician Global Assessment of disease activity at the last visit prior to the index date (one day following the baseline period).  Abbreviations: C4, complement 4; dsDNA, anti-double stranded DNA; MACE, major adverse cardiovascular event; SLE, systemic lupus erythematosus | | |

| **Table 3. Novel SLECRISK, American College of Cardiology/American Heart Association (ACC/AHA), Framingham Risk Score (FRS), Modified Framingham Risk Score (mFRS) Model Performances for Prediction of <7.5% (low risk) vs. ≥7.5% (moderate/high risk) One and Ten-Year Risks of MACE^1^ among 1,243 Patients with SLE at Baseline** | | | | |
| --- | --- | --- | --- | --- |
|  | **SLECRISK** | **ACC/AHA** | **FRS** | **mFRS** |
| Low Risk (<7.5%), n (%) | 673 (54.1) | 1074 (86.4) | 781(62.8) | 693 (55.8) |
| Moderate Risk (7.5-20%), n (%) | 456 (36.7) | 142 (11.4) | 313 (25.2) | 205 (16.5) |
| High Risk (>20%), n (%) | 114 (9.2) | 27 (2.2) | 149 (12.0) | 345 (27.8) |
| Sensitivity (95%CI) | 0.74 (0.65, 0.83) | 0.38 (0.28, 0.48) | 0.67 (0.57, 0.76) | 0.74 (0.65, 0.83) |
| Specificity (95%CI) | 0.56 (0.54, 0.59) | 0.88 (0.86, 0.90) | 0.65 (0.62, 0.68) | 0.58 (0.55, 0.61) |
| Positive Predictive Value (95%CI) | 0.12 (0.09, 0.14) | 0.20 (0.14, 0.26) | 0.13 (0.10, 0.16) | 0.12 (0.09, 0.15) |
| Negative Predictive Value (95%CI) | 0.97 (0.95, 0.98) | 0.95 (0.93, 0.96) | 0.96 (0.95,0.98) | 0.97 (0.95, 0.98) |
| c-statistic (95%CI) p-value for SLECRISK vs. Each Risk Score | 0.74 (0.69, 0.80) p= reference | 0.71 (0.65, 0.76) p=0.28 | 0.72 (0.67, 0.78) p=0.49 | 0.72 (0.67, 0.78) p= 0.51 |
| AIC | 592.18 | 617.81 | 595.78 | 595.35 |
| Hosmer Lemeshow Chi-square statistic (p-value) | 11.97 (0.15) | 17.58 (0.02) | 13.23 (0.10) | 14.41 (0.07) |
| Net Reclassification Index (95%CI) p-value for SLECRISK vs. Each Risk Score | - | 0.05 (-0.08, 0.19) p=0.42 | 0.003(-0.10, 0.10) p=0.95 | -0.006 (-0.11, 0.09) p=0.90 |
| Integrated discrimination improvement (95%CI) p-value for SLECRISK vs. Each Risk Score | - | 0.04 (0.01, 0.08) p=0.02 | 0.01 (-0.04, 0.06) p=0.81 | 0.001 (-0.06, 0.06) p=1.02 |
| 1. Definite (adjudicated) and probable MACE   Abbreviations: AIC, Akaike’s information criterion; AUC, area under the curve; ACC/AHA American College of Cardiology/American Heart Association; CI: confidence interval; FRS, Framingham Risk Score; mFRS, modified Framingham Risk Score. | | | | |

**Table 4. Comparison of Baseline Characteristics Between Moderate and High-Risk Patients (7.5% and greater) for Ten-Year Risks of MACE^1^ among 1,243 Patients with SLE at Baseline using the American College of Cardiology/American Heart Association Score vs. SLECRISK**

| **Baseline Characteristics*** | **SLECRISK (n=446)** | **ACC/AHA**  **(n=169)** | **P-value*** |
| --- | --- | --- | --- |
| Demographics, mean (SD) | | |  |
| Age | 41.3 (11.4) | 53.9 (17.6) | <0.0001 |
| Female, n (%) | 419 (94.0) | 145 (85.8) | 0.001 |
| CVD Traditional Risk Factors, mean (SD) | | |  |
| Total Cholesterol, mg/dL | 182.9 (59.5) | 191.8 (41.3) | 0.04 |
| LDL, mg/dL | 101.3 (30.0) | 107.5 (27.6) | 0.02 |
| SBP, mmHg | 121.2 (16.5) | 137.8 (22.7) | <0.0001 |
| DBP, mmHg | 75.9 (11.6) | 80.3 (13.5) | 0.0003 |
| Anti-Hypertensive, n (%) | 182 (40.8) | 94 (55.6) | 0.001 |
| Smoking, n (%) | 54 (12.1) | 40 (23.7) | 0.0004 |
| Diabetes, n (%) | 24 (5.4) | 35 (20.7) | <0.0001 |
| SLE Variables, mean (SD) | | |  |
| Mean SLE Duration (SD) | 15.0 (9.8) | 12.2 (11.2) | 0.005 |
| Lupus nephritis, n (%) | 198 (44.4) | 57 (33.7) | 0.02 |
| Autoantibodies, n (%) |  |  |  |
| Anti-dsDNA | 387 (86.8) | 118 (69.8) | <0.0001 |
| Anti-RNP | 252 (56.5) | 55 (32.5) | <0.0001 |
| Anti-Sm | 205 (46.0) | 49 (29.0) | 0.0001 |
| Anti-SSB/La | 186 (41.7) | 39 (23.1) | <0.0001 |
| Anti-Ro | 309 (69.3) | 78 (46.2) | <0.0001 |
| Any Antiphospholipid Ab | 131 (29.4) | 34 (20.1) | 0.02 |
| Lupus anticoagulant | 97 (21.8) | 11 (6.5) | <0.0001 |
| Anti-cardiolipin IgG | 137 (30.7) | 27 (16.0) | 0.0002 |
| Anti-β2 Glycoprotein I IgG | 46 (10.3) | 7 (4.1) | 0.01 |
| Anti-β2 Glycoprotein I IgM | 20 (4.5) | 2 (1.2) | 0.049 |
| Low C3, n (%) | 304 (68.2) | 68 (40.2) | <0.0001 |
| Low C4, n (%) | 270 (60.5) | 50 (29.6) | <0.0001 |
| Medications, n (%) |  |  |  |
| Mycophenolate | 89 (20.0) | 22 (13.0) | 0.046 |
| Azathioprine | 82 (18.4) | 15 (8.9) | 0.004 |
| Statin | 64 (14.4) | 38 (22.5) | 0.02 |
| *Only statistically significant variables are shown (p<0.05). Non-significant variables include pga1, pga2, Sm, SSB/La, antinuclear antibody, any antiphospholipid antibody, cardiolipin IgM, B2 IgM and IgG, steroids, hydroxycholoroquine, mycophenolate mofetil, cyclophosphamide, rituximab, cyclosporin, leflunomide, methotrexate, azathioprine, tacrolimus, intravenous immunoglobulin, belimumab, aspirin, warfarin, body mass index, total cholesterol, diastolic blood pressure, antihypertensive, sex, race/ethnicity, SLE duration, smoking, diabetes, %lupus nephritis   1. Definite (adjudicated) and probable MACE   Abbreviations: ACC/AHA, American College of Cardiology/American Heart Association; CVD, cardiovascular disease; HDL, high-density lipoprotein; SBP, systolic blood pressure; SLE, systemic lupus erythematosus | | | |

**Figure 1. Study Design.** We identified patients with SLE based using ICD9/10 codes and meeting ACR criteria without a history of major adverse cardiovascular event (MACE, myocardial infarction (MI), stroke, and cardiac death) from the electronic medical records (EMR). One-year baseline data including traditional CVD risk factors, demographics, and SLE-related clinical features were collected from the EMR at cohort enrollment. We allowed for a one-year extension period to collect covariate data that was missing from baseline. Ten-year follow-up for first MACE began at day +1 following the baseline period (index date). ICD-9/10 codes identified MACE adjudicated by medical record review by board-certified cardiologists.

**
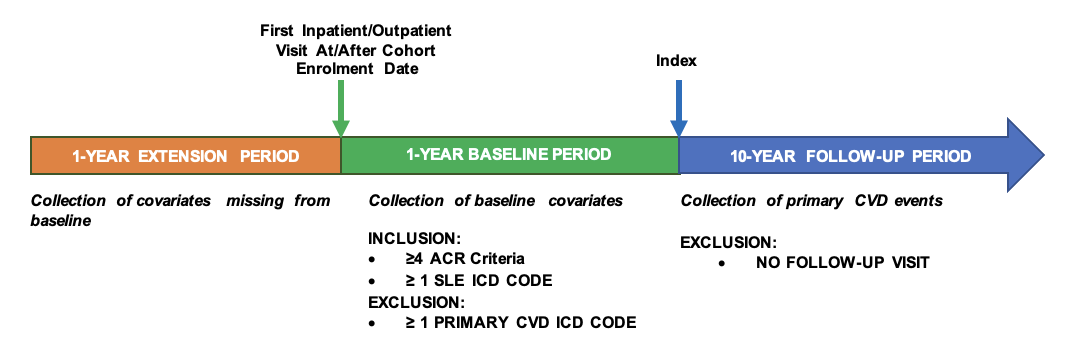
**

**Figure 2. Receiver Operating Curves at 10 years for novel SLECRISK compared to the Models of the American College of Cardiology/American Heart Association (ACC/AHA), Framingham Risk Score (FRS), Modified Framingham Risk Score (mFRS)**

**
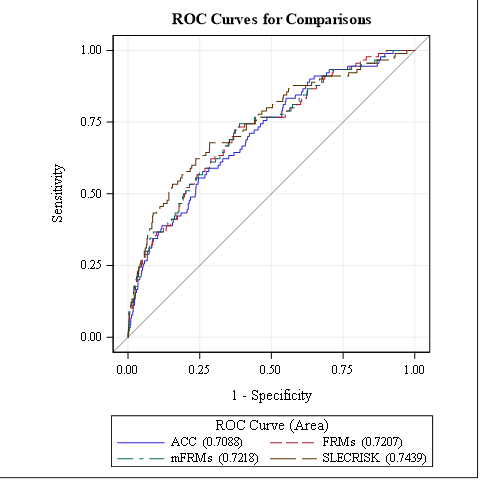
**

**Figure 3. Calibration plots for ten-year predictions for MACE.** X-axis is the deciles of the American College of Cardiology (ACC)/American Heart Association (AHA) (A), Framingham (B), and modified Framingham (C) scores. Y-axis is the actual probability (observed definite and probable MACE event) and predicted probability (risk score from the SLECRISK formula).

**SUPPLEMENTAL TABLES AND FIGURES**

**Supplemental Table 1. ICD-9/10 Codes for Myocardial Infarction (MI) and Strokes**

**PRIMARY ICD-10 CODES FOR MI**

| I21 | Acute myocardial infarction |
| --- | --- |
| I22 | Subsequent myocardial infarction |
| I23 | Complications after myocardial infarction |
| I24 | Other acute ischemic heart disease |
| I25 | Chronic ischemic heart disease |

**PRIMARY ICD-9 CODES FOR MI**

| 410 | Acute myocardial infarction |
| --- | --- |
| 411 | Other acute and subacute forms of ischemic heart disease. |
| 412 | Old MI |
| 414 | Other chronic ischemic heart disease |

**PRIMARY ICD-10 CODES FOR STROKE**

| I63 | Cerebral infarction |
| --- | --- |
| I64 | Stroke not specified as hemorrhage or infarction |

**PRIMARY ICD-9 CODES FOR STROKE**

| 434 | Occlusion of cerebral arteries including cerebral thrombosis and Cerebral embolism and unspecified cerebral artery occlusion |
| --- | --- |
| 436 | Acute, but ill-defined, cerebrovascular disease. |

**Supplemental Table 2. Physician Global Assessment Definitions**

| Score | Disease Activity | Definition |
| --- | --- | --- |
| 1 | Remission/Mild | Keyword in notes^2^: “remission”, “quiescent”, “burnt out”, “mild”, “low activity”  EULAR^1^: constitutional symptoms/mild arthritis/rash ≤9% BSA/PLTs 50-100 x 10^3^/mm^3^; SLEDAI≤6; BILAG C or ≤1 BILAG manifestation  Medications: on no SLE medication or low-dose anti-malarial, low-dose steroids, anti-malarial, NSAID, stable dose of DMARD |
| 2 | Moderate | Keyword in notes: “moderate”  EULAR^1^: RA-like arthritis/rash 9-18% BSA/cutaneous vasculitis ≤18% BSA; PLTs 20-50 x 10^3^/mm^3^; SLEDAI 7-12; ≥2 BILAG B manifestations  Medications: moderate dose steroids, biologics, multiple/escalating dose of DMARDs |
| 3 | Severe | Keyword in notes: “severe”  EULAR^1^: Major organ threatening disease (nephritis, cerebritis, myelitis, pneumonitis, mesenteric vasculitis; PLTs <20 x 10^3^/mm^3^, TTP-like disease or acute hemophagocytic syndrome; SLEDAI >12; ≥1 BILAG A manifestations  Medications: high dose/pulse steroids, cyclophosphamide, plasmapheresis |

^1^Fanouriakis, Antonis, et al. "2019 update of the EULAR recommendations for the management of systemic lupus erythematosus." Annals of the rheumatic diseases 78.6 (2019): 736-745.

| **Supplemental** **Table 3. Novel SLECRISK, American College of Cardiology/American Heart Association (ACC/AHA), Framingham Risk Score (FRS), Modified Framingham Risk Score (mFRS) Model Performances for Prediction of <10% (low risk) vs. ≥10% (moderate/high risk) One and Ten-Year Risks of Definite and Probable MACE among 1,243 Patients with SLE at Baseline** | | | | |
| --- | --- | --- | --- | --- |
|  | **SLECRISK** | **ACC/AHA** | **FRS** | **mFRS** |
| Low Risk (<10%), n (%) | 864 (69.5) | 1144 (92.0) | 891 (71.7) | 747 (60.1) |
| Moderate Risk (10-20%), n (%) | 265 (21.3) | 72 (5.8) | 203 (16.3) | 151 (12.2) |
| High Risk (>20%), n (%) | 114 (9.2) | 27 (2.2) | 149 (12.0) | 345 (27.8) |
| Sensitivity* (95%CI) | 0.64 (0.55, 0.74) | 0.27 (0.18,0.36) | 0.58 (0.48, 0.68) | 0.72 (0.63, 0.81) |
| Specificity* (95%CI) | 0.72 (0.70, 0.75) | 0.94 (0.92, 0.95) | 0.74 (0.71,0.77) | 0.63 (0.60, 0.65) |
| Positive Predictive Value* (95%CI) | 0.15 (0.12, 0.19) | 0.24 (0.16, 0.33) | 0.15 (0.11, 0.18) | 0.13 (0.10, 0.16) |
| Negative Predictive Value* (95%CI) | 0.96 (0.95, 0.98) | 0.94 (0.93, 0.96) | 0.96 (0.94,0.97) | 0.97 (0.95, 0.98) |
| c-statistic (95%CI) | 0.74 (0.69, 0.80) | 0.71 (0.65, 0.76) | 0.72 (0.67, 0.78) | 0.72 (0.67, 0.78) |
| AIC | 592.18 | 617.81 | 595.78 | 595.35 |
| Hosmer Lemeshow Chi-square statistic (p-value) | 11.97 (0.15) | 17.58 (0.02) | 13.23 (0.10) | 14.41 (0.07) |
| Net Reclassification Index (95%CI) p-value for SLECRISK vs. Each Risk Score | - | 0.16 (0.04, 0.28) p=0.01 | 0.06 (-0.06, 0.17) p=0.31 | 0.03 (-0.08, 0.13) p=0.62 |
| Integrated discrimination improvement (95%CI) p-value for SLECRISK vs. Each Risk Score | - | 0.04 (0.01, 0.08) p=0.02 | 0.01 (-0.04, 0.06) p=0.81 | 0.001 (-0.06, 0.06) p=1.02 |
| *Performance assessed using the using cut-off <10% (low risk) vs. ≥10% (moderate/high risk)  Abbreviations: AIC, Akaike’s information criterion; ACC/AHA American College of Cardiology/American Heart Association; CI: confidence interval; FRS, Framingham Risk Score; mFRS, modified Framingham Risk Score. | | | | |

**Supplemental Table 4. Beta-Coefficients for Predicting 10-year risk^1^ of Definite (Adjudicated MACE among 1243 Patients with SLE using SLECRISK and the American College of Cardiology/American Heart Association (ACC/AHA) Risk Score Alone**

| **Variable** |  | **Definite MACE only** |
| --- | --- | --- |
| ACC/AHA risk score only | | |
| ACC/AHA | Without SLE variables | 6.51 |
| SLECRISK | | |
| ACC/AHA | With SLE variables | 5.40 |
| SLE Disease Activity^2^ | Remission/mild vs. moderate/severe | 0.34 |
| SLE Disease Duration | Years | 0.04 |
| Serum Creatinine | mg/dL | 0.17 |
| Anti-dsDNA | Ever positive at baseline | 0.33 |
| Anti-RNP | Ever positive at baseline | 0.41 |
| Lupus Anticoagulant | Ever positive at baseline | 0.40 |
| Anti-Ro | Ever positive at baseline | 0.15 |
| Low C4 | Ever low at baseline | 0.51 |
| 1. Beta coefficients derived from Cox regression model estimating the hazard of developing MACE in ten years of follow-up.  2. Based on Physician Global Assessment of disease activity at the last visit prior to the index date (one day following the baseline period).  Abbreviations: C4, complement 4; dsDNA, anti-double stranded DNA; MACE, major adverse cardiovascular eventl SLE, systemic lupus erythematosus | | |

**Supplemental Table 5. Model performance comparing ACC/AHA, Framingham Risk Score, Modified Framingham Risk Score to SLECRISK using cut-off <7.5% (low risk) vs. 7.5% or greater (moderate/high risk) for Definite (adjudicated) MACE** **among 1,243 Patients with SLE at Baseline**

|  | **SLECRISK** | **ACC/AHA** | **FRS** | **mFRS** |
| --- | --- | --- | --- | --- |
| Sensitivity* (95%CI) | 0.76 (0.66, 0.85) | 0.38 (0.27, 0.49) | 0.66 (0.55, 0.77) | 0.73 (0.63, 0.83) |
| Specificity* (95%CI) | 0.56 (0.53, 0.59) | 0.88 (0.86, 0.90) | 0.65 (0.62, 0.67) | 0.58 (0.55, 0.60) |
| Positive Predictive Value* (95%CI) | 0.10 (0.07, 0.12) | 0.17 (0.11, 0.22) | 0.11 (0.08, 0.13) | 0.10 (0.07, 0.12) |
| Negative Predictive Value* (95%CI) | 0.97 (0.96, 0.99) | 0.96 (0.95, 0.97) | 0.97 (0.96, 0.98) | 0.97 (0.96, 0.98) |
| c-statistic (95%CI) | 0.75 (0.69, 0.81) | 0.71 (0.65, 0.77) | 0.71 (0.65, 0.77) | 0.71 (0.65, 0.77) |
| AIC | 517.81 | 536.84 | 518.61 | 521.77 |
| Hosmer Lemeshow Chi-square statistic (p-value) | 14.63 (0.07) | 16.97 (0.03) | 10.25 (0.25) | 9.22 (0.32) |
| Net Reclassification Index (95%CI) p-value for SLECRISK vs. Each Risk Score | - | 0.07 (-0.08, 0.21) p=0.36 | 0.03 (-0.09, 0.14) p=0.65 | 0.03 (-0.08, 0.14) p=0.59 |
| Integrated discrimination improvement (95%CI) p-value for SLECRISK vs. Each Risk Score | - | 0.03 (0.00, 0.07) p=0.04 | -0.002 (-0.06, 0.05) p=0.93 | 0.006 (-0.07, 0.06) p=0.85 |
| *Performance assessed using the using cut-off <7.5% (low risk) vs. 7.5% or greater (moderate/high risk)  Abbreviations: ACC/AHA, American College of Cardiology/American Heart Association; CI: confidence interval; FRS, Framingham Risk Score; mFRS, modified Framingham Risk Score. | | | | |

**Supplemental Table 6. Model performance comparing ACC/AHA, Framingham Risk Score, Modified Framingham Risk Score to SLECRISK using cut-off <10% (low risk) vs. 10% or greater (moderate/high risk) for Definite (Adjudicated) MACE among 1,243 Patients with SLE at Baseline**

|  | **SLECRISK** | **ACC/AHA** | **FRS** | **mFRS** |
| --- | --- | --- | --- | --- |
| Sensitivity* (95%CI) | 0.64 (0.53, 0.74) | 0.26 (0.16, 0.36) | 0.57 (0.45, 0.68) | 0.72 (0.61, 0.82) |
| Specificity* (95%CI) | 0.72 (0.69, 0.74) | 0.93 (0.92, 0.95) | 0.73 (0.71, 0.76) | 0.62 (0.59, 0.65) |
| Positive Predictive Value* (95%CI) | 0.12 (0.09, 0.16) | 0.19 (0.11, 0.27) | 0.12 (0.09, 0.15) | 0.11 (0.08, 0.13) |
| Negative Predictive Value* (95%CI) | 0.97 (0.96, 0.98) | 0.95 (0.94, 0.96) | 0.96 (0.95, 0.98) | 0.97 (0.96, 0.98) |
| c-statistic (95%CI) | 0.75 (0.69, 0.81) | 0.71 (0.65, 0.77) | 0.71 (0.65, 0.77) | 0.71 (0.65, 0.77) |
| AIC | 517.81 | 536.84 | 518.61 | 521.77 |
| Hosmer Lemeshow Chi-square statistic (p-value) | 14.63 (0.07) | 16.97 (0.03) | 10.25 (0.25) | 9.22 (0.32) |
| Net Reclassification Index (95%CI) p-value for SLECRISK vs. Each Risk Score | - | 0.15 (0.02, 0.28) p=0.02 | 0.06 (-0.07,0.19) p=0.39 | 0.03 (-0.09, 0.15) p=0.66 |
| Integrated discrimination improvement (95%CI) p-value for SLECRISK vs. Each Risk Score | - | 0.03 (0.00, 0.07) p=0.04 | -0.002 (-0.06, 0.05) p=0.93 | 0.006 (-0.07, 0.06) p=0.85 |
| *Performance assessed using the using cut-off <10% (low risk) vs. 10% or greater (moderate/high risk)  Abbreviations: ACC/AHA, American College of Cardiology/American Heart Association; CI: confidence interval; FRS, Framingham Risk Score; mFRS, modified Framingham Risk Score. | | | | |

**Supplemental Figure 1. Cohort Inclusion/Exclusion Criteria**
